# Case report: Manual carbon hemoperfusion for the treatment of meloxicam toxicity in a cat and suspected ibuprofen toxicity in a dog

**DOI:** 10.3389/fvets.2024.1395967

**Published:** 2024-09-18

**Authors:** Lauren E. Haire, Amber D. Vitalo, Ronald P. Gonçalves, Travis M. Lanaux

**Affiliations:** Department of Small Animal Clinical Sciences, University of Florida, Gainesville, FL, United States

**Keywords:** cats, dogs, non-steroidal anti-inflammatory drug, extracorporeal blood purification, hemoperfusion

## Abstract

Extracorporeal blood purification (ECBP) has become a popular treatment option for non-steroidal anti-inflammatory drug (NSAID) toxicity in small animals. However, challenges arise when using ECBP for small dogs and cats because the priming volume required by most machine-based ECBP platforms might be excessive, leading to cardiovascular instability if a blood prime is not used. This report describes the successful use of manual carbon hemoperfusion (MCHP) to reduce plasma meloxicam levels in a cat following an inadvertent overdose and its use in a dog following suspected ibuprofen ingestion. In both animals, MCHP reduced the circuit volume needed for ECBP from 125 mL with a machine-based therapeutic plasma exchange or 104 mL with an in-series carbon hemoperfusion on an intermittent hemodialysis platform to just 40–50 mL. In the cat, MCHP reduced plasma meloxicam levels by 44%, and in both animals, the use of MCHP in these pets was well-tolerated and safe. Due to pre-existing anemia, the cat required a blood transfusion but the dog did not. MCHP is technically simple and can be performed at any hospital with access to carbon filters and blood bank resources. This technique may represent a reasonable alternative to treat NSAID toxicities in animals that are too small for conventional extracorporeal decontamination methods using either machine-based platforms without using a blood prime or in locations where these machines are unavailable.

## Introduction

Non-steroidal anti-inflammatory drug (NSAID) exposure is a common veterinary toxicity (1, 2). Potential sequelae of NSAID toxicity include gastrointestinal signs (ulceration, vomiting, diarrhea, and melena), acute kidney injury (AKI), neurologic dysfunction, and hepatotoxicity (1). Traditional medical management includes gastrointestinal decontamination (for oral ingestions), administration of gastroprotectant medications, and intravenous (IV) fluid therapy (1, 3–5). Intravenous lipid emulsion (ILE) therapy is also described (6–9).

Recently, extracorporeal blood purification (ECBP) therapies have gained popularity in the treatment of NSAID toxicities in companion animals with successful mitigation of the effects of severe toxicoses (4, 5, 8–17). Therapeutic plasma exchange (TPE) has been reported to reduce plasma NSAID levels by 51–85.5% after a single session (9, 10, 12–14). Hemoperfusion has been less commonly reported in small animals but has successfully reduced plasma NSAID levels by 37–79%; all previous reports used an intermittent hemodialysis platform with an in-series dialyzer and older charcoal sorbents (15, 16). However, the circuit volumes for commercially available extracorporeal treatment systems in the United States are large. For example, the priming volume for commercially available membrane-based TPE in the United States is 125 mL ± 10%.^1^ Similarly, the minimum blood volume required to prime a neonatal IHD blood set with an in-series hemoperfusion column and low-efficiency dialyzer at the author’s institution is 104 mL.^2^^,^^3^ For smaller animals, ECBP would typically require a blood prime to prevent critical hypovolemia and a loss of tissue perfusion. Hemodialysis catheter placement is also technically more challenging in smaller patients, and hemodialysis and machine-based TPE both require costly equipment.

Here, we report the successful management of a meloxicam overdose in a cat and suspected ibuprofen ingestion in a dog using manual carbon hemoperfusion (MCHP) via a triple-lumen central line using a 40–50 mL circuit and without using a hemodialysis machine.

## Case 1

An 11-month-old neutered male domestic shorthair cat weighing 3.5 kg presented to the emergency service of a tertiary referral hospital for evaluation of a degloving injury. An initial temperature could not be obtained, but other vitals were within normal limits. On physical examination, the cat was found to be in moderate pain with a large degloving injury to the left pelvic limb with cutaneous myiasis along the wound margins. The remainder of the physical examination was unremarkable.

Following the initial sedated wound care, the cat was admitted to the ICU.^4^ The following day, therapy with meloxicam was initiated (0.1 mg/kg, SC, q24 h). Approximately 24 h after the initial dose, a high dose of meloxicam was inadvertently administered (0.48 mg/kg, SC). Immediately after the meloxicam overdose was administered to the cat, the error was noted and therapy with famotidine (1.2 mg/kg, IV followed by a CRI of 0.33 mg/kg/h, IV) and sucralfate (0.17 g/kg, PO, q8 h) was initiated in addition to previous treatments.

**Figure 1:**
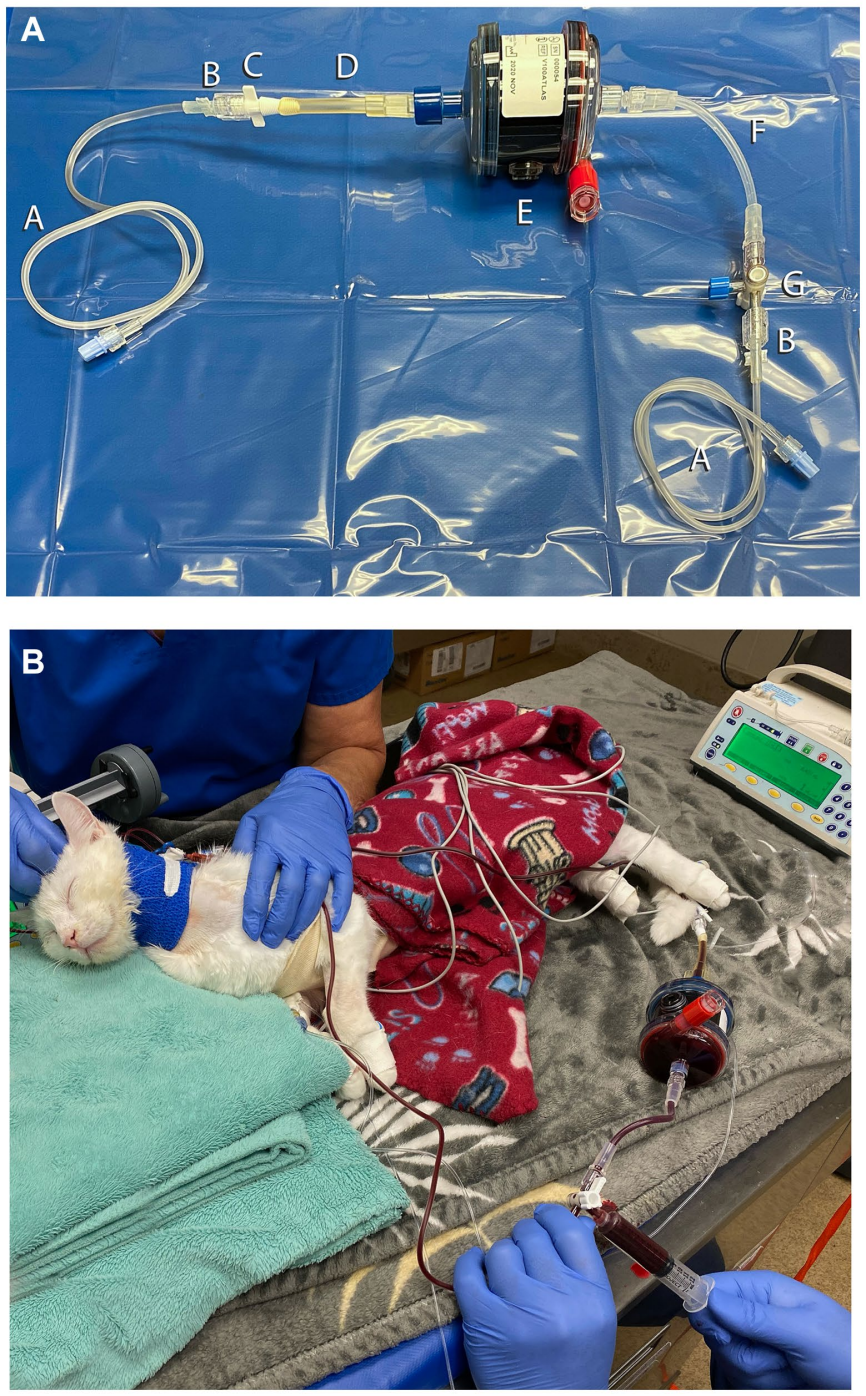
Circuit setup for manual carbon hemoperfusion in the cat (Case 1). **(A)** Circuit setup for Case 1. A, 53-cm IV extension set; B, Male–male adaptor; C, Christmas tree adaptor; D, cut portion of hemodialysis circuit; E, AimaLogic V100 Atlas Hemoperfusion Column; F, 7-cm IV extension set; G, 3-way stopcock. **(B)** Cat undergoing manual carbon hemoperfusion. A 12 mL syringe was connected to the stopcock to manually remove blood from the central line, push through the hemoperfusion column, and return the blood via a peripheral IV catheter to minimize access recirculation.

Approximately 8 h after the overdose, ECBP was elected. The cat was considered too small for machine-based TPE or carbon hemoperfusion (CHP) on an intermittent hemodialysis platform due to circuit volume requirements and the need for multiple units of blood to prime the machine. Manual TPE was considered too time-intensive, so it was decided to attempt MCHP. To facilitate central line placement, the cat was sedated with acepromazine (0.026 mg/kg, IV) and propofol (5.2 mg/kg, IV titrated to effect), and a 5.5-Fr, 8-cm, triple-lumen catheter was aseptically placed in the left external jugular vein using the modified Seldinger technique (18). A CHP column (ImmutriX V100 Atlas) (see text footnote 2) was selected, and a circuit was created by cutting part of the line of a neonatal hemodialysis circuit (see text footnote 4) and fitting the cut end onto the circuit with a Christmas tree adaptor (Figure 1). Other circuit components included two 53-cm extension sets, a 7-cm extension set, a three-way stopcock, two male–male adaptors, and a 12-mL syringe (Figure 1). The 12-mL syringe was used to push blood through the circuit, removing blood via the central line and returning blood via a peripheral IV catheter to minimize access recirculation. The column priming column was 40 mL, the created circuit was approximately 10 mL, and the entire circuit volume was approximately 50 mL. The circuit and hemoperfusion column were primed with 1 L of 0.9% saline that contained 5,000 u/L of unfractionated heparin, 500 mL of dextrose 5% in water (D5W), and a second liter of 0.9% saline per the CHP column manufacturer’s directions. The pet was administered unfractionated heparin to prevent clotting in the circuit or column (25 u/kg bolus followed by CRI of 25 u/kg/h, IV). Anticoagulation was monitored with activated clotting times (ACT) and titrated to maintain an ACT of 200–300 s (normal <120 s). Throughout MCHP, ECG, blood pressure, mentation, and respiratory rate, as well as electrolytes, blood glucose, and PCV and TS were monitored (Table 1). Before the MCHP treatment, the cat’s hematocrit was low (Table 1), thus one unit of type-matched packed red blood cells (9.3 mL/kg, IV) was transfused over 4 h before, during, and after the treatment.

**Table 1:** Venous blood gas results before, during, and after proposed MCHP on day 3 of treatment in Case 1.

Test	Result	Result	Result	Result	Result	Reference Interval
	Pre-MCHP	30 min in MCHP	60 min in MCHP	Post-MCHP	1-h Post-MCHP	
	Pre-transfusion				Post-transfusion	
pH	7.413			7.467	7.425	7.335–7.446
pvCO_2_	2.87 (21.5)			2.77 (20.8)	3.32 (24.9)	4.67–5.33 kPa (35–40 mmHg)
pvO_2_	9.65 (72.4)			6.27 (47.0)	6.07 (45.5)	4.27–8.27 kPa (32–62 mmHg)
SO_2_%	94.1			84.7	81.1	68–92%
Hct	22			20	27	40–52%
Hb	4.47 (7.2)			4.1 (6.6)	5.65 (9.1)	8.69–16.14 mmol/L (14–26 g/dL)
Na^+^	148 (148)			135.3 (135.3)	147.4 (147.4)	146–151 mmol/L (146–151 mEq/L)
K^+^	3.60 (3.60)			3.77 (3.77)	4.21 (4.21)	3.98–4.41 mmol/L (3.98–4.41 mEq/L)
Cl^−^	128.8 (128.8)			117.2 (117.2)	119.7 (119.7)	108.5–116.0 mmol/L (108.5–116.0 mEq/L)
Ca^2+^	0.63 (1.26)	0.56 (1.11)	0.54 (1.08)	0.57 (1.13)	0.60 (1.20)	0.59–0.68 mmol/L (1.18–1.35 mEq/L)
Glucose	10.9 (196)	ADL	ADL	ADL	8.16 (147)	4.83–6.16 mmol/L (87–111 mg/dL)
Lactate	0.07 (0.6)			0.09 (0.8)	0.08 (0.7)	0.04–0.17 mmol/L (0.4–1.5 mg/dL)
BUN	6.43 (18)			5.71 (16)	5.0 (14)	3.57–10.7 mmol/L (10–30 mg/dL)
Creatinine	106.1 (1.2)			88.4 (1.0)	70.7 (0.80)	70.7–132.6 μmol/L (0.8–1.5 mg/dL)
HCO_3_^−^	13.9 (13.9)			15.2 (15.2)	16.5 (16.5)	18–27 mmol/L (18–27 mEq/L)
PCV	23			24	29	30–45%
TS	6			5.8	6	68–83 g/L (6.8–8.3 g/dL)
Serum color	Clear			Clear	Clear	

MCHP, manual carbon hemoperfusion. Hb, hemoglobin; PCV, packed cell volume; TS, total solids. ADL, above detection limits.

A total of 1,171 mL of blood, or approximately 5.5 blood volumes, was processed over the treatment period of 60 min with an average blood flow rate of approximately 19.5 mL/min. The blood volume processed was measured by counting the number of syringes used to push the blood through the machine. At the end of the treatment, the circuit was flushed with 60 mL of 0.9% saline to return blood to the pet. Transient hyperglycemia and mild hypocalcemia were noted (Table 1), but the cat was not clinically affected by these derangements, and they resolved without intervention within 1 h of completion of the treatment. His anemia improved after completion of the transfusion (Table 1).

Following treatment, meloxicam was discontinued, and the patient was maintained on IV fluid therapy (LRS, 2.5 mL/kg/h, IV), famotidine (0.33 mg/kg/h, IV, CRI), and sucralfate (0.17 g/kg, PO, q8 h). He remained hospitalized for several days for continued wound management and monitoring. Treatment with famotidine and sucralfate was discontinued approximately 36 h and 5 days after MCHP, respectively. The cat was noted to be progressively anemic 5 days after MCHP (Table 2), suspected to be secondary to chronic loss from his large wound. A second unit of packed red blood cells (10.8 mL/kg, IV) was transfused to improve oxygen delivery and wound healing. On day 13 of treatment, the cat underwent partial wound closure. The cat was discharged 14 days after admission and 12 days after MCHP. No additional medications to treat meloxicam toxicity were prescribed to go home. Repeated bloodwork to assess renal function was not performed at follow-up visits over the next several weeks for wound care. However, the cat remained asymptomatic for NSAID toxicosis and appeared to have no adverse effects of MCHP treatment.

**Table 2:** Serial laboratory values before, during, and after MCHP in Case 1.

Value	Results Day 1	Results Day 4	Results Day 5	Results Day 7	Results Day 8	Results Day 13	Reference interval
pH	7.364	7.412	7.438	7.464		7.389	7.335–7.446
pvCO_2_	4.06 (30.5)	3.67 (27.5)	3.81 (28.6)	3.51 (26.3)	80 (8.0)	4.13 (31.0)	4.67–5.33 kPa (35–40 mmHg)
pvO_2_	41.7 (5.56)	59.3 (7.91)	39.9 (5.32)	50.0 (6.67)		33.4 (4.45)	4.27–8.27 kPa (32–62 mmHg)
SO_2_%	73	90.3	74.7	86.2		64.8	68–92%
Hct	39	27	28	25		32	40–52%
Hb	7.94 (12.8)	5.59 (9.0)	5.65 (9.1)	5.15 (8.3)		6.64 (10.7)	8.69–16.14 mmol/L (14–26 g/dL)
Na^+^	150.6 (150.6)	147.4 (147.4)	149.2 (14.9.2)	146.9 (146.9)		154.9 (154.9)	146–151 mmol/L (146–151 mEq/L)
K^+^	4.58 (4.58)	3.96 (3.96)	3.84 (3.84)	3.4 (3.4)		4.08 (4.08)	3.98–4.41 mmol/L (3.98–4.41 mEq/L)
Cl^−^	120.3 (120.3)	120.3 (120.3)	119.6 (119.6)	117.4 (117.4)		122.3 (122.3)	108.5–116.0 mmol/L (108.5–116.0 mEq/L)
Ca^2+^	0.60 (1.20)	0.62 (1.24)	0.64 (1.27)	0.61 (1.21)		0.66 (1.32)	0.59–0.68 mmol/L (1.18–1.35 mEq/L)
Glucose	5.94 (107)	10.1 (182)	8.99 (162)	7.77 (140)		4.88 (88)	4.83–6.16 mmol/L (87–111 mg/dL)
Lactate	0.11 (1.0)	0.08 (0.7)	0.12 (1.1)	0.07 (0.6)		0.06 (0.5)	0.04–0.17 mmol/L (0.4–1.5 mg/dL)
BUN	8.57 (24)	6.43 (18)	7.50 (21)	5.71 (16)		7.14 (20)	3.57–10.7 mmol/L (10–30 mg/dL)
Creatinine	97.3 (1.1)	88.4 (1.0)	88.4 (1.0)	88.4 (1.0)		88.4 (1.0)	70.7–132.6 μmol/L (0.8–1.5 mg/dL)
HCO_3_^−^	17.5 (17.5)	17.7 (17.7)	19.5 (19.5)	19.0 (19.0)		18.9 (18.9)	18–27 mmol/L (18–27 mEq/L)
PCV	40			24	27	33	30–45%
TS	76 (7.6)			72 (7.2)	80 (8.0)	80 (8.0)	68–83 g/L (6.8–8.3 g/dL)
Serum color	Clear			Clear	Clear	Clear	

MCHP, manual carbon hemoperfusion. performed on day 3 of treatment; Hb, hemoglobin; PCV, packed cell volume; TS, total solids.

Immediately before and after the MCHP treatment as well as 12 h and 24 h after MCHP, plasma samples were obtained from the central line before the hemoperfusion column for meloxicam quantification via performance liquid chromatography^5^ (Table 3). Blood samples were collected into lithium-heparin blood tubes and centrifuged, and the heparinized plasma was stored at −80°C prior to submission. Clearance was calculated using the formula:

$$K_{p}=V\times \frac{\ln\left(C_{0}/C_{\mathrm{end}}\right)}{t\times BW}\text{,}$$

**Table 3:** Plasma meloxicam concentrations before and after MCHP in Case 1.

Time of sample	Plasma concentration (μg/mL)	Percent reduction from the previous timepoint	Percent reduction from baseline	Calculated clearance from previous timepoint (mL/kg/min)
Pre-MCHP, 8 h post-administration	1.35	–	–	–
Post-MCHP, 9 h post-administration	0.75	44.4%	44.4%	2.65
12 h post-MCHP, 21 h post-administration	0.67	10.7%	50.4%	0.04
24 h post-MCHP, 33 h post-administration	0.44	34.3%	67.4%	0.16

MCHP, manual carbon hemoperfusion.

where *K_p_* is the plasma clearance of meloxicam (mL/kg/min); *V* is the total volume of distribution of meloxicam (mLs); *C*_0_ is the initial serum concentration (μg/mL); *C_end_* is the final serum concentration (μg/mL); *t* is the duration (min); and *BW* is the patient body weight (kg) (17). MCHP reduced plasma meloxicam concentrations in this cat by 44.4% and provided a plasma clearance of 2.65 mL/kg/min (Table 3).

## Case 2

A 1-year-old spayed female mixed breed dog weighing 2.61 kg was presented to a satellite clinic of the emergency and critical care service of a tertiary referral hospital for possible ingestion of approximately 50,200 mg tablets of ibuprofen in the 4–5 h prior to presentation. The maximum possible ingestion for this pet was 3,703 mg/kg. The owner attempted to induce vomiting with hydrogen peroxide at home but was unsuccessful. No significant previous medical history was reported. On presentation to the clinic, the vitals of the dog were normal and her physical examination was unremarkable. A blood gas analysis was performed, and PCV and TS were monitored and revealed mild hyperlactatemia but were otherwise unremarkable (Table 4). Vomiting was induced with apomorphine (0.03 mg/kg, IV), and the pet vomited brown partially digested food and pieces of plastic, but no obvious tablets were noted. She was administered maropitant (1 mg/kg, IV) and activated charcoal without sorbitol^6^ (10 mL/kg, PO). Due to concern for possible massive ingestion and severe NSAID toxicity, the dog was referred to the main campus of the tertiary referral hospital for further care.

**Table 4:** Blood gas values before, during, and after proposed MCHP in Case 2.

Test	Result	Result	Result	Reference interval
	Pre-MCHP	45 min in MCHP	Post-MCHP	
pH	7.36	7.259	7.325	7.335–7.446
pvCO_2_	3.08 (23.1)	5.65 (42.4)	5.09 (38.2)	4.67–5.33 kPa (35–40 mmHg)
pvO_2_	7.71 (57.8)	4.05 (30.4)	6.28 (47.1)	4.27–8.27 kPa (32–62 mmHg)
SO_2_%	86	37.8	74.1	68–92%
Hct	42	31.5	37.7	40–52%
Hb	9.87 (15.9)	6.33 (10.2)	7.63 (12.3)	8.69–16.14 mmol/L (14–26 g/dL)
Na^+^	143 (143)	147 (147)	148 (148)	146–151 mmol/L (146–151 mEq/L)
K^+^	3.95 (3.95)	3.80 (3.80)	3.20 (3.20)	3.98–4.41 mmol/L (3.98–4.41 mEq/L)
Cl^−^	114 (114)	121 (121)	120 (120)	108.5–116.0 mmol/L (108.5–116.0 mEq/L)
Ca^2+^	0.62 (1.23)	0.48 (0.95)	0.59 (1.18)	0.59–0.68 mmol/L (1.18–1.35 mEq/L)
Glucose	5.33 (96)	8.66 (156)	9.16 (165)	4.83–6.16 mmol/L (87–111 mg/dL)
Lactate	0.29 (2.6)	0.34 (3.1)	0.17 (1.5)	0.04–0.17 mmol/L (0.4–1.5 mg/dL)
BUN	5.36 (15)	–	–	3.57–10.7 mmol/L (10–30 mg/dL)
Creatinine	61.9 (0.70)	35.4 (0.40)	38.0 (0.43)	70.7–132.6 μmol/L (0.8–1.5 mg/dL)
HCO_3_^−^	13.22 (13.22)	17.2 (17.2)	19.4 (19.4)	18–27 mmol/L (18–27 mEq/L)
PCV	43%		34%	30–45%
TS	53 (5.3)		65 (6.5)	68–83 g/L (6.8–8.3 g/dL)
Serum color	Clear		Lipemic	

MCHP, manual carbon hemoperfusion. Hb, hemoglobin; PCV, packed cell volume; TS, total solids.

On presentation to the main hospital, the dog was mildly tachycardic (heart rate 180 bpm), but her other vitals were normal and her physical examination was unremarkable. She was administered maropitant (1 mg/kg, IV), IV fluid therapy (LRS, 2.9 mL/kg/h), pantoprazole (1 mg/kg, IV, q12 h), and IV ILE therapy (1.5 mL/kg bolus followed by a CRI of 0.25 mL/kg/min for 60 min). Two other larger dogs from the home were treated with machine-based TPE, which is the standard of care for NSAID toxicity at the author’s institution when available due to the high protein binding of these drugs. During the ILE administration, it was decided to attempt ECBP for this dog due to the potentially toxic effects of ingesting even a single tablet (76 mg/kg). ILE was discontinued; the total volume administered was not recorded. Similar to the previous case, this dog was not considered a good candidate for machine-based TPE due to her small size. Manual TPE was considered but rejected due to the length of time required to complete the treatment. Machine-based CHP was rejected out of concern for the patient’s small size, and MCHP was elected due to the small circuit volume requirement.

**Figure 2:**
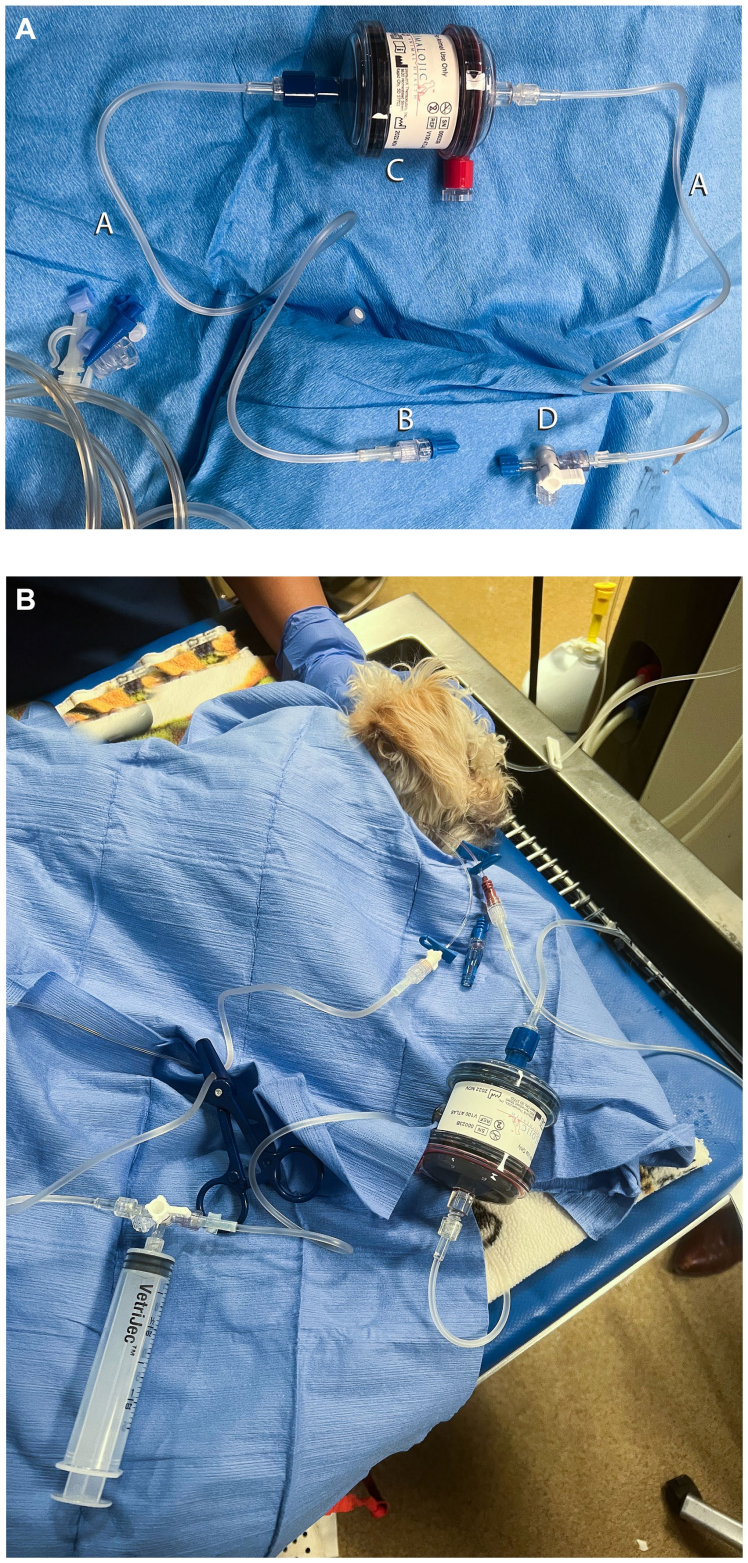
Circuit setup for manual carbon hemoperfusion in the dog (Case 2). **(A)** Circuit setup in Case 2. A, 53 cm IV extension set; B, Male–male adaptor; C, AimaLogic. V100 Atlas Hemoperfusion Column; D, 3-way stopcock. The male Luer lock end of the extension set was connected to the DIN on the venous side of the column, and the other end of the extension set was connected to a central venous catheter with a male–male adaptor. The air release port was used instead of the access or arterial DIN connection because that is how the circuit was used in previous cases at the author’s institution. The effects on blood flow and column adsorption are unknown. In future cases, the access DIN connection could be used to prevent such uncertainty. **(B)** Dog undergoing manual carbon hemoperfusion. A 12-mL syringe was connected to the stopcock to manually remove blood from the more distal white port of the central line, push through the hemoperfusion column, and return the blood via the more proximal brown port to minimize access recirculation.

To facilitate central line placement, the dog was sedated with butorphanol (0.2 mg/kg, IV) and dexmedetomidine (2 mcg/kg, IV). A 5.5Fr x 8 cm triple-lumen catheter was placed in the right jugular vein via the modified Seldinger technique. A CHP column (ImmutriX v100 Atlas) was selected, and a circuit was created with three 53-cm IV extension sets, a stopcock, a 12-mL syringe, and two male-to-male adaptors (Figure 2). The column priming volume was 40 mL, the circuit was approximately 8 mL, and the entire circuit volume including the column was approximately 48 mL. The circuit was primed, and anticoagulation was provided and monitored in the same manner as the cat in Case 1. A 12-mL syringe connected to the stopcock was used to push blood through the circuit, using the white port for access and the brown port for blood return to minimize access recirculation. During the treatment, sedation was maintained with an additional dexmedetomidine bolus (2 mcg/kg, IV) and butorphanol CRI (0.2 mg/kg/h). Throughout MCHP, ECG, blood pressure, mentation, and respiratory rate were monitored. Her electrolytes, blood glucose, and PCV were also monitored in the same manner similar to that of Case 1 (Table 4).

The patient experienced transient hypertension, hypotension, mild hypothermia, and regurgitation during MCHP. Active warming was provided to correct hypothermia. Hypotension resolved after slowing the blood flow rate and administration of a crystalloid bolus (LRS, 10 mL/kg, IV). Moderate hypocalcemia was noted at the time of regurgitation and corrected with a single bolus of 0.5 mL/kg calcium gluconate IV, diluted 1:1, and administered over 10–15 min. The pet was also administered ondansetron (0.5 mg/kg, IV). The continuous flow was provided in 12 mL aliquots for 90 min. The estimated blood volume processed was 4,320 mL or 18 blood volumes. At the end of the 90-min treatment, the apparatus was flushed with 60 mL of 0.9% sterile saline to return blood to the pet. After the session, venous blood gas analysis and PCV/TS were repeated (Table 4). The patient regurgitated once more, and ondansetron and maropitant were repeated at the previous doses. ILE therapy was not continued after the session due to persistent gross lipemia.

After treatment, the patient was maintained on IV fluids (LRS, 2.9 mL/kg/h, IV), N-acetylcysteine (140 mg/kg bolus followed by 70 mg/kg, IV, q6 h), pantoprazole (1 mg/kg, IV, q12 h), maropitant (1 mg/kg, IV, q2 h), ondansetron (0.5 mg/kg, IV, q8 h), cholestyramine (2 g, PO, q8 h), and metoclopramide CRI (2 mg/kg/day, IV). The dog was monitored in-hospital for signs of NSAID toxicosis. No gastrointestinal side effects were noted. A serum chemistry was performed the following day, which showed no evidence of AKI or hepatotoxicity (Table 5). She was prescribed lansoprazole (1 mg/kg, PO, q12 h) to be administered at home for an additional 5 days and discharged to the owner’s care the day after admission. At follow-up 24 h after discharge, the dog was reportedly doing well with no outward signs of NSAID toxicity or adverse effects related to MCHP treatment.

**Table 5:** Serum chemistry values, 24 h after MCHP in Case 2.

Value	Result	Reference interval
CK	200	49–244 U/L
AST	29	16–53 U/L
ALT	55	23–93 U/L
GGT	<3	6–10 U/L
ALP	84	7–116 U/L
Total Bilirubin	3.4 (0.2)	1.7–6.8 μmol/L (0.1–0.4 mg/dL)
Glucose	5.72 (103)	4.33–6.88 mmol/L (78–124 mg/dL)
Cholesterol	4.14 (160)	4.97–8.97 mmol/L (192–340 mg/dL)
Triglycerides	0.63 (56)	0.42–2.66 mmol/L (37–235 mg/dL)
Albumin	28.5 (2.85)	26.2–39.1 g/L (2.62–3.91 g/dL)
Globulins	21 (2.1)	18–40 g/L (1.8–4.0 g/dL)
Total protein	49 (4.9)	50–74 g/L (5.0–7.4 g/dL)
BUN	2.86 (8)	2.5–9.64 mmol/L (7–27 mg/dL)
Creatinine	46.0 (0.52)	53.0–132.6 μmol/L (0.6–1.5 mg/dL)
Sodium	140.7 (140.7)	141.9–150.6 mmol/L (141.9–150.6 mEq/L)
Potassium	3.0 (3.0)	3.8–5.0 mmol/L (3.8–5.0 mEq/L)
Chloride	111.6 (111.6)	107.8–117.1 mmol/L (107.8–117.1 mEq/L)
Bicarbonate	19 (19)	16–24 mmol/L (16–24 mEq/L)
Calcium	2.25 (9.0)	2.25–2.74 mmol/L (9.0–11.0 mg/dL)
Phosphorous	0.94 (2.9)	0.71–1.55 mmol/L (2.2–4.8 mg/dL)
Magnesium	0.66 (1.6)	0.70–0.99 mmol/L (1.7–2.4 mg/dL)
PCV	33	35–57%
TS	52 (5.2)	60–80 g/L (6.0–8.0 g/dL)
Serum color	Clear	

MCHP, manual carbon hemoperfusion. CK, creatine kinase; AST, aspartate aminotransferase; ALT, alanine aminotransferase; GGT, gamma-glutamyl transferase; ALP, alkaline phosphatase; PCV, packed cell volume; TS, total solids.

Before and immediately after the MCHP treatment, plasma samples were obtained similarly to Case 1 and submitted for ibuprofen quantification via performance liquid chromatography. Both samples were below the limits of detection.

## Discussion

Here, we report the use of MCHP to treat meloxicam overdose in a cat and suspected ibuprofen toxicosis in a dog. In general, CHP can remove toxins with low-to-moderate V_d_ (<1 L/kg), up to 40,000 Da, and very high protein binding (>99%) via adsorption (19). Though uncommonly, this technique has been reported in dogs to treat carprofen and ibuprofen toxicities. Previous reports have all utilized an intermittent hemodialysis platform with an in-series dialyzer (15, 16, 19). Ours is the first report of CHP in the cat, the first report of CHP to treat meloxicam toxicity, and the first report of an adapted novel MCHP therapy without a machine platform, or in-series dialyzer in both species.

Previous reports using charcoal hemoperfusion columns to treat NSAID toxicity in dogs have achieved 34–49.5% reduction and clearance of 1.4–2.1 mL/kg/min for carprofen after 1 h of treatment with an in-series dialyzer (16) and up to 79% reduction for ibuprofen after 6 h of treatment with an in-series dialyzer, replacing the hemoperfusion column halfway through treatment (15). In the cat reported here, plasma meloxicam levels were reduced by 44.4% during the 1-h MCHP session, compared to 10.7% over the subsequent 12 h and 34.3% reduction over an additional 12 h. The clearance with MCHP was 2.65 mL/kg/min, which was 16-66X the calculated intrinsic clearance in this cat over the subsequent 12–24 h. In the dog, plasma levels of ibuprofen were below the limits of detection, thus ibuprofen clearance provided by this technique could not be calculated. The dog was included in this report due to the novel circuit developed for MCHP without the need to cut and adapt hemodialysis circuit lines.

The animals included in this report were treated for 60–90 min, based on the CHP column manufacturer recommendations and previous reports in dogs that revealed column saturation after 1–2 h (15, 16). A previously reported case achieved up to 79% reduction after changing the column during the treatment (15). Thus, greater reduction levels in the cat could have been achieved potentially by exchanging the column and continuing treatment.

Previous reports of CHP have all utilized a machine-based IHD platform and performed treatment with the charcoal column and a dialyzer in series to prevent hypoglycemia and hypocalcemia and provide warming to the extracorporeal blood (15, 16). In the present cases, the circuits were primed with dextrose solution per the CHP column manufacturer’s directions, preventing hypoglycemia even without the use of an in-series dialyzer. It is unclear why the cat treated here became markedly hyperglycemic as this has not occurred in other cases treated with MCHP at the author’s institution, but no adverse effects were detected, and the hyperglycemia resolved without intervention. It is speculated that dextrose in the packed red blood cell transfusion or possibly residual D5W in the circuit could have contributed to the hyperglycemia. Mild, transient ionized hypocalcemia was noted in both animals. The cat was not clinically affected, and supplementation was not required. The dog experienced nausea and regurgitation, but both hypocalcemia and nausea resolved with the administration of a calcium gluconate bolus. External warming was provided with a heat support device to prevent significant hypothermia.

In addition to hypoglycemia and hypocalcemia, thrombocytopenia, leukopenia, and a decrease in coagulation factors are well-known potential complications of hemoperfusion (18, 19). In previous reports in small animals, thrombocytopenia (15, 16) and clinically significant coagulopathy (15) have been reported, though these reports utilized older, activated charcoal sorbents. In the current patients in whom more biocompatible carbon sorbents were used, platelet and white blood cell counts were not measured immediately before or after the treatment, thus it is unknown whether there were decreases in any cell lines. No significant bleeding, petechiation, or clinical signs of leukopenia or thrombocytopenia were noted. The cat was noted to have thrombocytopenia the day prior to discharge, but clumping was observed and the true platelet count could not be estimated.

In both animals, manual hemoperfusion also allowed the use of a smaller, triple-lumen catheter, which is readily available at most emergency hospitals and avoided the need for a larger-bore hemodialysis catheter required to achieve adequate blood flow using traditional IHD or TPE platforms. Additionally, the manual circuit reduced the extracorporeal blood volume to 40–50 mL from circuit volumes greater than the pets’ volumes with traditional machine-based ECBP techniques. A blood prime was not required for the dog using the manual circuit, while it most certainly would have been required to perform machine-based ECBP safely. The cat was administered a blood transfusion during the MCHP session due to pre-existing anemia, which is similar to performing a blood prime. Although the authors would not have performed a blood prime in this patient if the anemia were resolved, we cannot conclude that a blood prime is not required in cats using this technique. A second transfusion was administered during the cat’s care due to recurrent anemia 5 days after the MCHP treatment. The authors attributed the progressive anemia to repeated blood sampling and blood loss during ongoing wound care but cannot definitively exclude MCHP as a contributing factor.

Other limitations of MCHP as described in this report include a lack of precise control over blood flow rate and the lack of safety sensors and alarms to detect potentially fatal complications such as clotting in the circuit or air emboli. Future treatment directions could include the use of a blood transfusion pump to control flow rates more precisely. Additionally, a limitation of MCHP, as described in Case 1, is the need to cut hemodialysis lines to create a closed circuit, which could pose risks for maintaining sterility. As that connection was not a locking connection, it could also become dislodged during the session and lead to significant blood loss and a loss of sterility. The circuit setup utilizing male-to-male adaptors in Case 2 was included in this report as it mitigated those risks. Using DIN to Luer lock connections would mitigate those risks; these connectors would provide an even more secure connection between the extension tubing and the hemoperfusion column.

Both animals presented here were concurrently treated with standard medical management (IV fluids, gastric acid reducers, and GI protectants) as is standard practice when ECBP techniques are utilized to treat NSAID toxicity (4, 5, 8–16). No clinical signs of NSAID overdose, such as GI hemorrhage or acute kidney injury, were noted in either pet, although the relative contributions of MCHP versus standard medical management to the mitigation of clinical signs of toxicosis are unknown. Prospective studies would be required to recommend MCHP over standard medical management, TPE, or traditional machine-based CHP with an in-series dialyzer to treat NSAID toxicity in small animals. However, in the cases presented here, MCHP was well-tolerated and technically simple. In the cat, it provided a significant reduction in plasma meloxicam levels without significant complications, while also allowing the application of ECBP to animals considered too small for treatment with traditional machine-based platforms.

## Data Availability

The original contributions presented in the study are included in the article/supplementary material, further inquiries can be directed to the corresponding author.

## Footnotes

^1^Prismaflex™ TPE 2000 Set, Baxter Healthcare Corp, Deerfield, IL.

^2^ImmutriX V100 Atlas hemoperfusion column, AimaLogic Animal Health, Rapidtown, SD.

^3^Gambro™ low weight-low volume blood set (Baxter Healthcare Corp, Deerfield, IL) with and Hemoflow™ F3 dialyzer (Fresenius Kabi USA LLC, Lake Zurich, IL).

^4^Sedation protocols and other treatments administered to the cat for wound management are available upon request.

^5^Clinical Pharmacology Laboratory, North Carolina State University, Raleigh, NC.

^6^ToxiBan^®^, containing 10% MedChar™ and 6.25% kaolin, Lloyd Inc., Shenandoah, IA.
